# An adjustable predictive score of graft survival in kidney transplant patients and the levels of risk linked to *de novo* donor-specific anti-HLA antibodies

**DOI:** 10.1371/journal.pone.0180236

**Published:** 2017-07-03

**Authors:** Aurélie Prémaud, Matthieu Filloux, Philippe Gatault, Antoine Thierry, Matthias Büchler, Eliza Munteanu, Pierre Marquet, Marie Essig, Annick Rousseau

**Affiliations:** 1 INSERM, U850, Limoges, France; 2 Univ. Limoges, UMR_S850, Limoges, France; 3 CHU Limoges, Service d’immunologie et immunogénétique, Limoges, France; 4 CNRS, CRIBL, UMR 7276, Limoges, France; 5 CHU Tours, Service Néphrologie–et Immunologie Clinique, Tours, France; 6 CHU Poitiers, Service de Néphrologie-Hémodialyse-Transplantation rénale, Poitiers, France; 7 CHU Limoges, Service de néphrologie, dialyse-transplantations, Limoges, France; 8 CHU Limoges, Service de pharmacologie, toxicologie et pharmacovigilance, Limoges, France; University of Toledo, UNITED STATES

## Abstract

Most predictive models and scores of graft survival in renal transplantation include factors known before transplant or at the end of the first year. They cannot be updated thereafter, even in patients developing donor-specific anti-HLA antibodies and acute rejection.We developed a conditional and adjustable score for prediction of graft failure (AdGFS) up to 10 years post-transplantation in 664 kidney transplant patients. AdGFS was externally validated and calibrated in 896 kidney transplant patients.The final model included five baseline factors (pretransplant non donor-specific anti-HLA antibodies, donor age, serum creatinine measured at 1 year, longitudinal serum creatinine clusters during the first year, proteinuria measured at 1 year), and two predictors updated over time (*de novo* donor-specific anti-HLA antibodies and first acute rejection). AdGFS was able to stratify patients into four risk-groups, at different post-transplantation times. It showed good discrimination (time-dependent ROC curve at ten years: 0.83 (CI95% 0.76–0.89).

## Introduction

Scoring systems that predict survival outcome after kidney transplantation can help physicians improve risk stratification among recipients and make the best therapeutic decision for a patient who develops *de novo* donor-specific anti-human leucocyte antigen (HLA) antibody (DSA). Serum creatinine (Scr) and estimated glomerular filtration rate (GFR) are not sufficiently reliable predictors for long-term risk of graft loss or patient death [1]. In the last decade, predictive models of graft survival based on large panels of data collected in the donor [2], in the recipient before transplantation [3], and/or in the first year post-transplantation [4,5] have been proposed. A limitation of these models is that they do not take into account the onset of adverse events over time, which modify graft outcome. In particular, these models never consider the impact of the development of *de novo (dn)*DSA beyond one year post-transplantation on graft outcome, although this has been demonstrated to be strongly associated with graft loss through antibody-mediated rejections [6,7]. All the studies focusing on the impact of the development of de novo DSA on graft outcome have concluded that post–transplant DSA monitoring could improve prediction of individual risk for kidney allograft loss [5,8]. The previously proposed tools were globally validated in patient cohorts but they often lost their predictive power in small patient subgroups with specific risks of graft failure, i.e. the patients who need them most.

Development of a graft failure risk score is most often based on Cox’s proportional hazards models (eventually with time-dependent covariates) to identify predictive risk factors [4,9,10] Random survival forest (RSF) modeling is an alternative non-parametric method based on an ensemble tree method for the analysis of right censored survival data [11]. RSF was found able to identify complex interactions among multiple variables and performed better than traditional cox proportional hazard model [12]. Other advantages of RSF are (i) insensitivity to noise brought by missing values or error data [11] and (ii) inclusion of an internal validation process [11]. Thus, RSF has been used in several risk models in cardiology [13] and oncology [14,15]. A conditional scoring system may be more appropriate than the addition of weights as derived from Cox model if the impact of a risk factor is different, whether or not it is associated with other factors. Finally, a prognostic tool that can be updated with comorbidity onset may be more powerful [16].

The objective of the present study was to build (using RSF) and validate a new conditional risk-scoring system of graft failure up to ten years post-transplantation, taking into account onset of emerging risks over time such as development of *dn*DSA. Our score highlights the impact of renal function during the first year and the evolution of the risk of graft loss with the onset of *dn*DSA and acute rejection.

## Methods

This study adheres to the Declaration of Istanbul.

### Database

**Fig 1:**
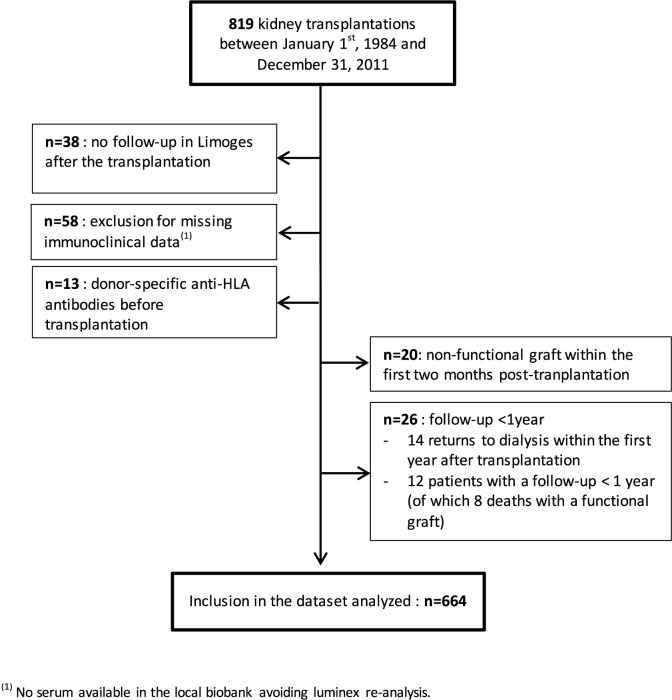
Flowchart showing selection of renal transplant patients.

Of the 819 transplantations performed at the University Hospital of Limoges (France) between december 1984 and december 2011, 664 were included in the primary cohort (development database). A flow-chart showing patient selection is shown in Fig 1. All 664 transplants studied came from heart-beating deceased donors and had a follow-up of at least one year after transplantation. The maintenance immunosuppressive regimen consisted mainly of one calcineurin inhibitor (cyclosporine or, since 2001, tacrolimus) associated with azathioprine (until 1996) or mycophenolate mofetil (after 1996) and corticosteroids (generally stopped between 3 and 6 months post-transplantation). All patients received induction therapy. Patient outcome was known for each patient at the date of the last follow-up. Death was considered as a censored event when the recipient died with a functioning graft. When graft function was not known on the exact date of death, the date of the last biological assessment before death was then considered as the censoring time. Usually, graft function was recorded a few days before death. When patients died because of graft loss, death was considered as a graft failure.

Donor, recipient and graft characteristics were collected from the CRISTAL register (from the French public agency “Agence de la Biomédecine”). Samples for immunological analysis were available in the local biobank, declared to the Ministry of Health (N° DC-2010-1074). At the time of transplantation, DNA was systematically extracted from the cells of the spleen or lymph node of the donor. DNA (storage 4°C) and cells (storage nitrogen) were preserved for each donor. Sera of the patients were systematically preserved at -20°C.

The study database was approved by the French Informatics and Liberty National Commission (CNIL, registration number 1795293).

### Anti-HLA antibodies screening

Anti-HLA-A, -B, -C, -DP, -DQ, -DR antibodies were screened and identified using Luminex® solid-phase assay (One Lambda LABScreen assays) in samples collected before transplantation and routinely after transplantation (three, six, twelve months, once every year thereafter, and whenever clinically indicated). Results were expressed as median fluorescence intensity (MFI). MFI >1000 was considered positive. All sera tested using the Complement Dependent Cytotoxicity method prior to the availability of Luminex® technology in our center (2007), were re-analyzed using Luminex®. As DQ, DP and C HLA typing was not previously systematically performed in our center, a molecular DNA typing of donor and recipient was performed in case of detection by Luminex® of an anti-HLA-C, -DQ or -DP antibody during the survey. This procedure allowed to determine the specificity (donor-specific or non donor-specific) of the anti-HLA antibody and to avoid bias in the determination of DSA. DSA diagnosis prior to renal transplantation was an exclusion criterion for transplantation in our center. Patients in whom the Luminex® reanalysis identified presence of DSA before transplantation (n = 13) were excluded from the database studied.

### Cluster analysis of serum creatinine over the first year post-transplantation

Homogeneous subgroups of trajectories of serum creatinine measured within the first year post-transplantation were identified by a clustering method based on k-means, specifically designed to analyze longitudinal data and implemented in the ‘kml’ R-package (version 1.1.3) [17]. This method does not require any assumption regarding the shape of the serum creatinine-time curves, contrary to model-based methods which fit the trajectories with a specific model (e.g. linear, polynomial or exponential). The optimal number of clusters was selected using the statistical criterion proposed by Calinski and Harabasz [18].

### Identification of factors predictive of graft survival

The impact of the following variables was investigated on graft survival: (i) donor characteristics (age, cause of death—cardiac, stroke or traumatic injuries-); (ii) recipient demographic variables (age at time of transplantation, gender); (iii) transplantation characteristics [time period of transplantation (i.e. 1984–1993, 1994–2003 or 2004–2011), cold ischemia time, previous kidney transplantation(s)]; (iv) immunological variables (HLA-A, HLA-B and HLA-DR mismatches, pre-transplant anti-HLA antibodies, source of anti-HLA alloimmunization (i.e. previous transplantation, pregnancy, blood transfusion), occurrence of *de novo* donor-specific and/or non-donor-specific anti-HLA antibodies (*dn*DSA and *dn*NDSA, respectively) with the date of the first diagnosis; (v) biological variables [repeated measurements of serum creatinine (μM) over the first year post-transplantation, proteinuria (g/L) at one year post-transplantation]; (vi) clinical variables (initial renal disease, date of first acute rejection diagnosis, date of return to dialysis, date of end of follow-up); and (vii) immunosuppressive drugs administered. Patient ethnicity was not recorded since it is not authorized by French law.

RSF analysis was performed to select and rank the most predictive covariates of graft failure using the date of transplantation as time origin [11]. RSF was implemented in the ‘randomForestSRC’ R-package (version 2.0.0). Briefly, a RSF was generated by creating 1000 trees, each tree built on a randomly selected bootstrap sample (using 63% of the original data) using a randomly selected subset of covariates. Each bootstrap sample excluded, on average, 37% of the data, which were reserved for a test set called “out-of-bag” data (OOB). RSF evaluated the change in prediction error attributable to each covariate. The prediction error (i.e. the percentage of patients misclassified) was assessed with the Harrell’s concordance index (Harrell’s c-index) using OOB data [19]. The c-index was computed using an OOB set constructed with the 1000 OOB datasets provided by the 1000 bootstrap samples used in growing the forest. The OOB prediction error is defined as 1 minus Harrell’s c-index [11]. The prediction error ranges between 0 and 1, where a value of 0.5 corresponds to a prediction no better than random guessing and a value of 0 reflects perfect accuracy. The parameter “nsplit” used to specify random splitting was fixed at 3. The predictive performance of the studied variables was evaluated by their “variable importance” (VIMP), calculated by RSF. VIMP measures the change in prediction error for a forest grown with or without this variable.

Variables selection was successively done by (1) fitting data by RSF and ranking all available variables and (2) iteratively fitting RSF by removing at each iteration a variable from the bottom of the positive variable importance ranking list. The minimal combination of variables leading to the smallest “out-of-bag” prediction error rate, assessed by the Harrell’s c-index, was selected.

A conditional survival tree [20] was subsequently drawn from the whole original dataset, using the most predictive variables selected from RSF [‘party’ (version 1.0–21) R-package].

### Prediction of graft failure

Score calculations were derived from both the VIMP sourced from the final RSF model and the conditional survival tree. The weight of each variable (i.e. each risk factor) was based on the ratio between its VIMP and the VIMP of the last predictive variable retained. A same value of weight was allocated for variables split at the same tree-depth in the conditional survival tree. The weighted risk score was calculated by adding the weights of the different risk factors within each branch of the conditional survival tree. This strategy led to a score for each patient subgroup identified at each terminal node of the conditional survival tree. Time-dependent receiver operating characteristic (ROC) curves with area under the curve (AUC) for censored survival data were used to evaluate the discrimination of the developed score. Additional weights were attributed for variables not selected in the conditional survival tree but highly associated with graft survival in the RSF analysis, provided their inclusion improved the ROC AUC. The weight of a factor could be increased if it allowed maximization of the ROC AUC at ten years post-transplantation. The predictive performance of the developed score was evaluated by time-dependent sensitivity, specificity, positive predictive value (PPV) and negative predictive value (NPV) with their standard error, all estimated at several cutpoints, i.e. for different threshold score values and for different times after transplantation. Therefore, ‘timeROC’ (version 0.2) R package was employed using the Kaplan-Meier estimator of the censoring distribution. Baseline (i.e. including variables available at one year post-transplantation) and adjusted (i.e. adding variables collected after one year post-transplantation) scores were also compared using time-dependent ROC AUC.

### External validation

External validation of the developed score was performed in patients transplanted between 2002 and 2010 in two independent French transplantation centers (CHU Tours n = 706; CHU Poitiers n = 190). As in the development cohort, patients with pre-transplant DSA were excluded. All anti-HLA antibodies screenings were performed using Luminex®. The validation database (Astre database) was approved by the CNIL (Authorization number DR-2012-518).

Validation procedure included: recalculation of the Scr clusters considering the external database only, calculation of the individual scores using the developed scoring system, determination of the time-dependent ROC AUC at ten years post-transplantation and calibration based on Hosmer-Lemeshow goodness-of-fit test adapted for survival data [21]. The calibration evaluation consisted in comparing numbers of patients with graft failure expected and observed in the validation cohort using the calculation of the numbers of events based on Kaplan-Meier survival estimates which was by proposed by D’Agostino-Nam [22]. In a first step, the number of graft failures observed in the validation cohort in different time-intervals ([0–2[, [2–4[, [4–6[, [6–8[, [8–10] years after transplantation) were calculated for each risk group as the product n_i_(*1-KM*_*i*_*(t)*) where *KM*_*i*_ is the Kaplan-Meier survival estimate at a fixed time t for group_i_ and n_i_ the number of observations in group_i._ The survival probabilities expected in the validation cohort were calculated using the Kaplan-Meier estimates obtained in the development cohort. With this test, the p value has to be higher than 0.05.

### Statistical analyses

The study used a conditional approach to determine a patient risk-stratification with several (>2) levels of risk of graft failure. An estimated 616 patients were needed for a power of 80% and a two-sided significance level of 5%. Based on published data and expert opinion, we assumed (i) a 10-years free graft failure survival of 82% in the studied population of renal transplant patients and (ii) that kidney function one year after the transplantation and occurrence of de novo DSA over time will be major discriminant parameters to classify the patients in the different risk levels. We hypothesized that 25% of the studied patients would have an impaired renal function one year after transplantation (i.e a serum creatinine concentration higher than 1.8 mg/dL (160 μmol/L) [1]) resulting in 10-years free graft failure survival being decreased to 70%. In agreement with Wiebe et al. [23] we hypothesized cumulative incidence of de novo DSA of 15% resulting in a 10-years free graft failure survival decreased to 60% and to 40% in the groups with a serum creatinine concentration lower than 160 μmol/L and higher than 160 μmol/L, respectively. Acute rejection being a known major risk factor of graft failure in patients with DSA [24], we considered that around 33% of the patients with DSA would have developed acute rejection and that their graft survival would be reduced to 25%.

Comparison between categorical data was done using the Pearson chi-square test or the exact Fisher test. Normally distributed data were analyzed by Anova and the parametric t-test, whereas nonparametric tests (Kruskall-Wallis and Mann–Whitney tests respectively) were used otherwise. Kaplan-Meier analysis was used to assess graft survival (graft loss, i.e. return to dialysis). Graft survival in different patient subgroups was compared using the log rank test.

Statistical analyses were performed with MedCalc for Windows, version 14.10.2. (MedCalc Software, Ostend, Belgium) and R version 2.15.1 (www.R-project.org). The R packages are freely available through the Comprehensive R Archive Network distribution system (http://cran.r-project.org).

## Results

### Development database

The characteristics of the studied kidney transplants are listed in Table 1.

**Table 1:** Kidney transplant characteristics of the development and validation databases.

	Development Database (n = 664)	Validation Database (n = 896)	
		Tours	Poitiers
Total number of transplants	664	706	190
Duration of follow-up (years)	6.4 (± 3.3)	7.4 (± 2.3)	7.9 (± 1.2)
Functional renal grafts at 10 years post-transplantation	202 (30.4%)	171 (24.2%)	31 (16.3%)
Recipient gender (M/F)	405/259	NA	NA
Recipient age (years)	49 (± 14) (15–77)*	NA	NA
Donor age (years)	44 (± 16)	51 (± 17)	48 (± 15)
HLA A Mismatch	1.2 (± 0.7)	NA	NA
HLA B Mismatch	1.5 (± 0.6)	NA	NA
HLA DR Mismatch	1.2 (± 0.7)	NA	NA
First transplantation	608 (91.6%)	NA	NA
Pretransplant NDSA	105 (15.8%)	151 (21.4%)	34 (17.9%)
Serum creatinine at M12 (μM)	139 (± 71)	136 (± 49)	131 (± 40)
Proteinuria at M12 (g/L)	0.18 (± 0.50)		0.19 (± 0.45)
Proteinuria at M12 (g/24h)		0.63 (± 1.96)	0.37 (± 0.80)
Return to dialysis	69 (10.4%)	116 (16.4%)	22 (11.6%)
Death with a functional graft (censured data)	60 (9.0%)	NA	NA
*de novo* NDSA	142 (21.3%)	NA	NA
Median (range) time to onset (years)	3.02 (0.02–10)		
*de novo* DSA	62 (9.3%)	113 (16%)	41 (21.6%)
Median (range) time to onset (years)	3.92 (0.02–9.83)	2.94 (0.79–5.04)	3.12 (1.95–5.1)
Patients with onset of *dn*DSA in the first year after transplantation	11 (17.7%)	35 (31.0%)	7 (16.7%)
First acute rejection episode	137 (20.6%)	219 (31%)	48 (25.3%)
Median (range) time to onset (years)	0.26 (0.01–9.27)	0.15 (0.04–0.35)	0.25 (0.03–0.40)

Data are n (%), mean (± SD) or median (range)

*4 patients under the age of 18 years (3 of them aged 17 years)

DSA = donor-specific anti-HLA antibodies. NDSA = non-donor-specific anti-HLA antibodies. M12 = month 12 post-transplantation. NA Not Appropriate

During the whole study period, 137 patients have been treated for a first acute rejection among them 122 (89%) were biopsy proven. One hundred nine first rejections occurred during the first year post-transplantation. Borderline rejection was evidenced in 36 patients and T-Cell mediated rejection (TCMR) in 105 patients, Antibody-mediated rejection (ABMR) in 14 patients and mixed (ABMR + TCMR) in three patients. Only two patients displayed ABMR criteria on a biopsy done before the definition of ABMR in the Banff classification.

During follow-up, *dn*DSA were present in 62 patients. The median time to *dn*DSA diagnosis was significantly lower in patients who exhibited pretransplant NDSA than in patients who did not (1.42 *vs* 4.87 years, p = 0.0012). Sixty-four percent of patients with *dn*DSA (n = 39) had class II antigens, 34% (n = 21) had class I and 2% (n = 2) had both class I and II antigens. Nearly all patients who developed *dn*DSA after transplantation had previously (n = 19) or concomitantly (n = 36) developed *dn*NDSA. Except for one patient who presented *dn*DSA transiently (i.e., detected at 1.6 years after transplantation and absent at subsequent screenings), DSA remained persistent at all screenings following the first detection. Thirteen patients with *dn*DSA returned to dialysis, including six within the year following the diagnosis of *dn*DSA (median 1.04 years, range: 0.03–4.46). Eleven out the 17 patients with ABMR on histology had developed *dn*DSA.

**Fig 2:**
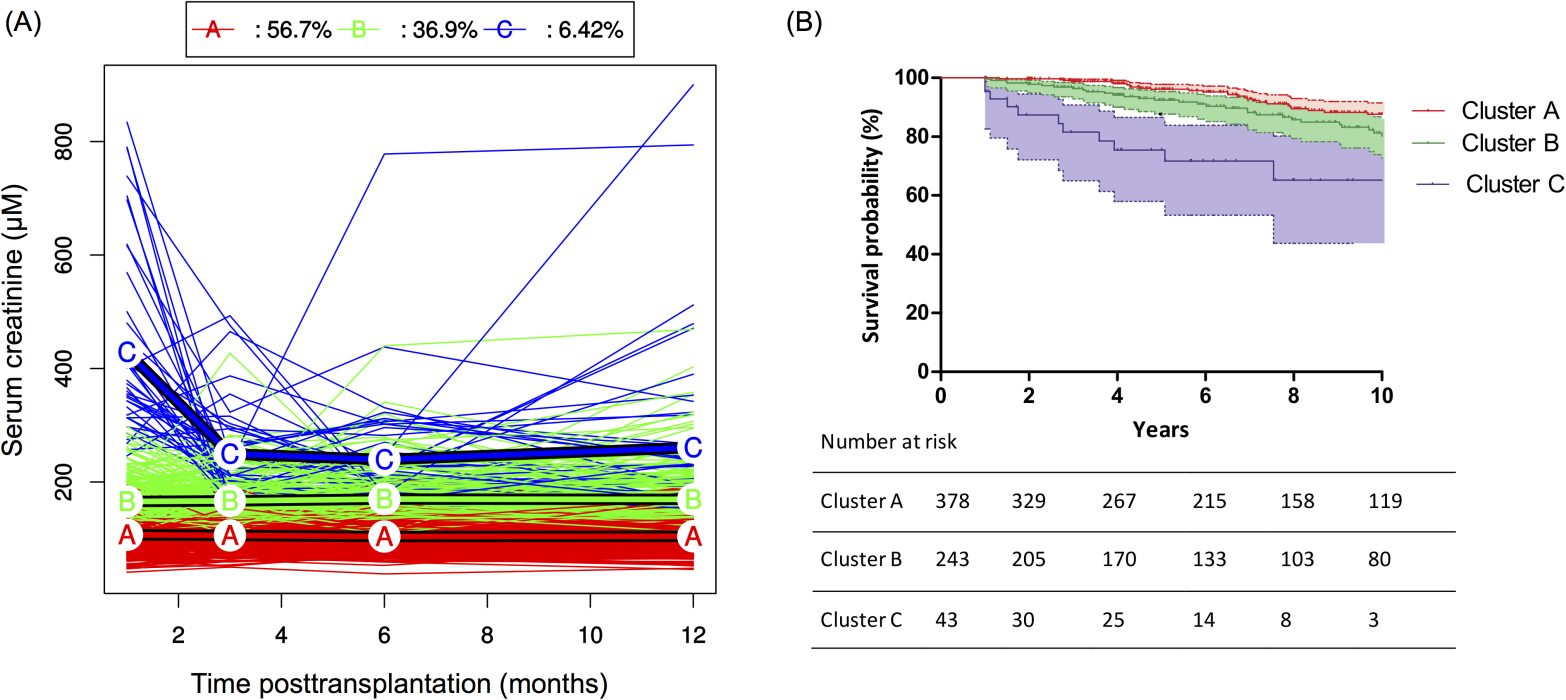
Serum creatinine clusters. (A) Mean trajectories of serum creatinine resulting from k-means for the longitudinal data clustering method superposed with individual profiles over the first 12 months post-transplantation. (B) Kaplan-Meier estimates (±95% confidence intervals) of graft survival according to the first-year creatinine profile cluster. The free graft failure survival was significantly associated with clusters (log-rank test, p<0.0001).

Scr profiles over the first year post-transplantation were best partitioned in three clusters (Fig 2). Graft survival after transplantation was significantly different in these three subgroups (p<0.0001) (Fig 2). The percentage of donors over 60 years of age increased from cluster A to C (29 [7.7%], 57 [23.4%], and 19 [44.2%], respectively, p<0.0001). The mean cold ischemia time was significantly higher in cluster C than in clusters A and B, p = 0.034). No cold ischemia time lower than 12 hours was observed in cluster C.

### Identification of factors predictive of graft survival after the first year post-transplantation

**Fig 3:**
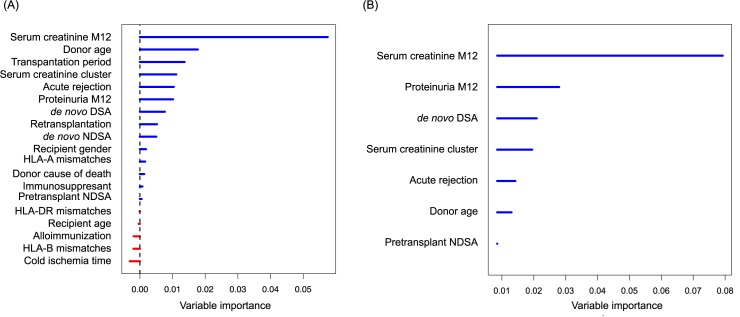
Out-of-bag data variable importance values obtained by random survival forest analysis. (A) full model and (B) final model. The log-rank splitting rule was used.

The classification of the variables according to their out-of-bag importance in the full RSF model is illustrated in the Fig 3. The best model was obtained using the log rank splitting rule with 1000 trees with a Harrell’s Concordance error rate of 21% (standard deviation 0.2%) (Fig 3). This final model included five baseline variables (pretransplant NDSA, donor age, Scr measured at 12 months post-transplantation (ScrM12), Scr clusters, proteinuria measured at M12 (ProtM12)), and two predictors which could be updated during the follow-up of the graft (onset of *dn*DSA and first acute rejection whatever the time of onset after transplantation).

**Fig 4:**
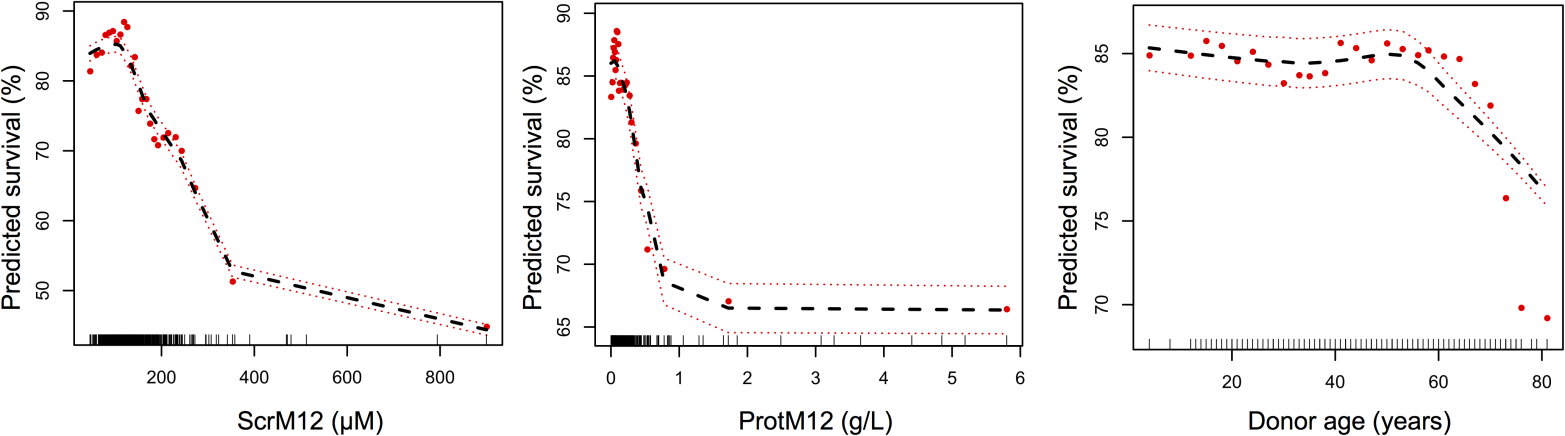
Partial plots for the continuous variables retained in the random survival forest analysis as predictors of graft failure. The vertical axis represents the predicted survival at 10 years for a given predictor, after adjusting for all other predictors. Points indicate partial values and dashed lines are ±2 standard error bars.

The partial plots of graft survival, predicted in the RSF analysis using the retained continuous variables (after adjusting for all other predictors) showed decreased survival when donor age exceeds 60 years, and very steep survival curves when ScrM12 >150 μM, so that small increments in ScrM12 would result in large survival declines (Fig 4).

### Adjustable graft failure score (AdGFS) for prediction of graft survival

**Fig 5:**
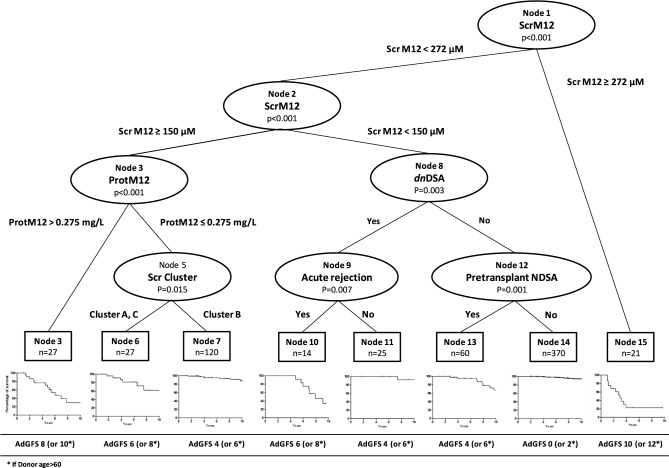
Conditional inference tree applied for graft survival with predicted Kaplan-Meier curves in the terminal nodes. The tree was obtained using recursive partitioning for censored response in a conditional inference framework implemented in ‘party’ R-package.

A scoring system was constructed using conditional survival tree analysis, with nodes corresponding to the variables selected in the final RSF model. The tree identified height terminal nodes, corresponding to height patient subgroups (Fig 5). The hierarchical order of the variables in predicting graft survival provided by the conditional survival tree was in accordance with the variable ranks obtained by RSF analysis.

**Fig 6:**
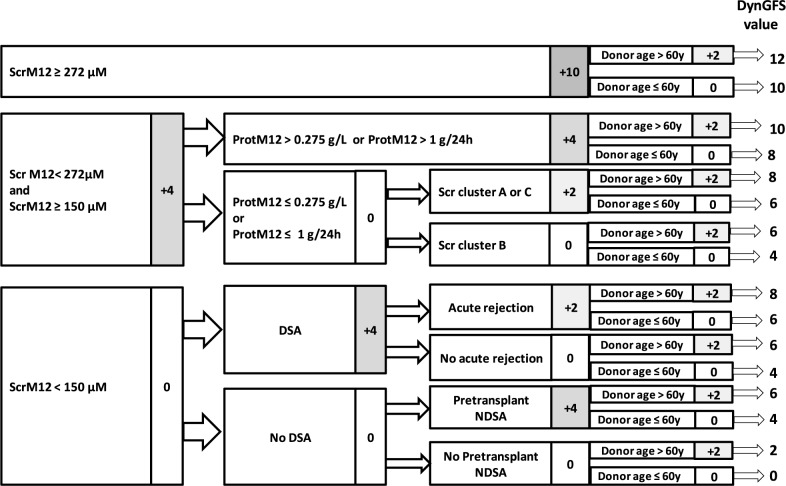
Scoring system for computing AdGFS values. ScrM12 = serum creatinine at 12 months post-transplantation. ProtM12 = proteinuria at 12 months post-transplantation. Scr = serum creatinine. *dn*DSA = *de novo* donor-specific anti-HLA antibodies. NDSA = non donor-specific anti-HLA antibodies.

Our scoring system, named AdGFS (Adjustable Graft Failure Score), is shown in Fig 6. AdGFS outperformed the baseline score including predictors available at one year after transplantation (time-dependent ROC AUC at ten years: 0.83 (CI95% 0.76–0.89) *vs* 0.75 (CI95% 0.68–0.82), p = 0.0075). Taking into account onset of *dn*DSA and first acute rejection developed over time, after one year post-transplantation improved survival prediction beyond 5 years post-transplantation (p = 0.0244).

**Fig 7:**
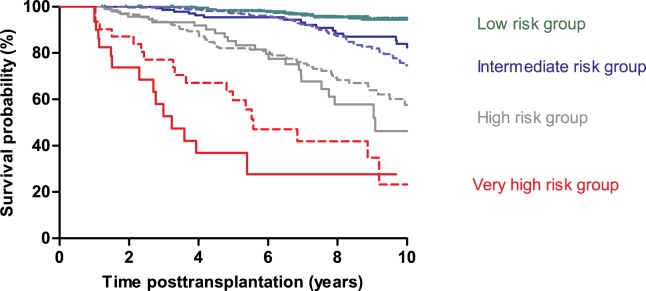
Comparison of Kaplan-Meier graft survival curves for the four risk groups namely low-, intermediate-, high-, and very high- risk of graft loss in the development dataset (solid lines) and in the external validation dataset (dashed lines). Patients were partitioned according to the calculated score value: low risk (0), intermediate risk (2 or 4), high risk (6 or 8), and very high risk (10 or 12). Graft survival in the development and validation datasets did not differ within each of the four risk groups.

AdGFS values are reported for each patient subgroup in Fig 5. Table 2 presents, for the different cutpoints of AdGFS values, the performance characteristics of graft survival prediction at different post-transplantation times. For example, a patient with low score (AdGFS = 2) has a probability of graft survival up to 10 years post-transplantation of approximately 94.5% (NPV). Onset *dn*DSA during the follow-up increased the score value (adjusted score = 6) and led to a probability of graft loss of 64.9% at 8 years and 83.6% at 10 years post-transplantation (PPV) (Table 2). Probabilities of graft survival lower than 20% (PPV > 80%) at ten years post-transplantation were obtained for score values of 6 and more. Risk groups were defined according to the AdGFS value: low risk (0), intermediate risk (2–4), high risk (6–8), and very high risk (10–12). Ten years graft survival was significantly different between these four risk groups (p< 0.0001) (Fig 7).

**Table 2:** Performance characteristics of adjustable graft failure score (AdGFS) for cutpoints 0, 2, 4, 6, 8, 10 and for different times over 10 years post-transplantation.

Cutoff point (c)	Number of positive tests (>c)	Number of negative tests (≤c)	Censored post-transplantation time (years)	Se (se_Se)%	Sp (se_Sp)%	PPV (se_PPV) %	NPV (se_NPV)%
0	292	365	2	100 (0)	59.7 (2.1)	4.3 (1.3)	100 (0)
	299	358	4	95.7 (4.2)	60.9 (2.3)	11.1 (2.1)	99.6 (0.4)
	303	354	6	85.7 (5.9)	66.2 (2.6)	17.9 (2.9)	98.1 (0.8)
	309	348	8	80.7 (5.5)	69.2 (2.9)	29.1 (3.8)	95.8 (1.4)
	314	343	10	79.4 (5.4)	72.5 (3.2)	35.9 (4.5)	94.8 (1.5)
2	264	393	2	100 (0)	63.2 (2.1)	4.6 (1.4)	100 (0)
	271	386	4	91.9 (5.5)	64.6 (2.3)	11.7 (2.3)	99.3 (0.5)
	275	382	6	83.3 (6.2)	68.5 (2.5)	18.5 (3.0)	97.9 (0.9)
	282	375	8	79.3 (5.6)	69.9 (2.9)	29.2 (3.9)	95.5 (1.4)
	288	369	10	78.3 (5.4)	73.1 (3.2)	36.0 (4.5)	94.5 (1.5)
4	120	537	2	100 (0)	85.4 (1.5)	11.0 (3.2)	100 (0)
	122	535	4	75.7 (8.6)	87.0 (1.6)	23.0 (4.6)	98.5 (0.6)
	125	532	6	70.6 (7.4)	91.0 (1.6)	40.2 (6.2)	97.3 (0.8)
	130	527	8	58.5 (6.7)	95.0 (1.4)	64.9 (7.4)	93.6 (1.4)
	134	523	10	53.7 (6.5)	97.7 (1.0)	83.6 (7.2)	91.6 (1.6)
6	62	595	2	90.9 (8.7)	93.3 (1.1)	19.8 (5.7)	99.8 (0.2)
	62	595	4	68.2 (9.2)	94.3 (1.1)	37.9 (7.2)	98.3 (0.6)
	62	595	6	56.6 (8.0)	96.0 (1.1)	55.2 (8.4)	96.2 (1.0)
	62	595	8	39.4 (6.4)	97.7 (1.0)	73.0 (9.0)	91.1 (1.6)
	62	595	10	33.1 (5.7)	98.4 (0.9)	80.7 (9.5)	88.4 (1.9)
8	31	626	2	62.8 (14.7)	97.5 (0.7)	31.1 (10.0)	99.3 (0.4)
	31	626	4	50.4 (9.7)	98.4 (0.6)	62.6 (10.9)	97.4 (0.7)
	31	626	6	34.2 (7.5)	99.1 (0.5)	77.6 (11.0)	94.6 (1.1)
	31	626	8	19.9 (4.9)	99.6 (0.4)	89.1 (10.0)	88.8 (1.7)
	31	626	10	16.7 (4.2)	100.0 (0.0)	100 (0)	86.1 (1.9)
10	9	648	2	17.6 (11.4)	99.1 (0.4)	26.2 (16.2)	98.5 (0.5)
	9	648	4	26.5 (8.7)	100 (0)	100 (0)	96.3 (0.8)
	9	648	6	16.3 (5.8)	100 (0)	100 (0)	93.3 (1.2)
	9	648	8	9.5 (3.5)	100 (0)	100 (0)	87.5 (1.7)
	9	648	10	7.9 (3.0)	100 (0)	100 (0)	84.9 (1.9)

Time post-transplantation was defined as the duration between the date of transplantation and the time point where graft failure prediction was made. The test was considered as positive when AdGFS score > cutpoint and negative when score was ≤ cutpoint. Time dependent sensitivity (Se), Specificity (Sp) Positive Predictive Value (PPV) and Negative Predictive Value (NPV) were computed with standard error (se) at the six given cutpoints: 0 and 2, 4, 6, 8, 10 for different censored post-transplantation times. AdGFS could be calculated in 657 patients, 7 patients were secondarily excluded due to missing data.

### External validation of AdGFS

Table 1 reports the characteristics of the patients. Graft survival within each risk group was similar in the development and external validation datasets (Fig 7). The accuracy of the score at predicting graft failure remained high in the validation dataset, with a time-dependent ROC AUC of 0.79 (CI 95% 0.74–0.84) at ten years after transplantation. Results of calibration evaluation of AdGFS in the external dataset were good: observed numbers of patients with graft failure were close to the expected numbers using the AdGFS risk groups ((χ_2_ = 2.39, p = 0.30) (Table 3).

**Table 3:** Goodness-of-fit test for external validation of the AdGFS score.

Risk group	Number of patients with graft failure		Number of patients without graft failure	
	Observed	Expected	Observed	Expected
Low (0)	14	14.9	314	313.1
Intermediate (2 or 4)	47	53.8	286	279.2
High (6 or 8)	57	58.3	146	144.7
Very high (10 or 12)	18	14.8	14	17.2

Data refers to the number of patients. Chi-squared = 2.39 (p = 0.30) with 2 degrees of freedom. The number of patients with graft failure expected in the validation cohort for the four different risk groups was calculated using the Kaplan-Meier survival estimates obtained in the development cohort.

## Discussion

In the present work, we developed and externally validated a conditional and adjustable predictive score (named AdGFS) of long-term kidney graft failure including pre-transplantation, early post-transplantation predictors and two factors collected all along the patients’ follow-up: onset of *dn*DSA and first acute rejection episodes. All the items included in the score are available everywhere in the day-to-day clinical surveillance of the patients. This score can be calculated from one year post-transplantation and updated all along the evolution of the graft depending on the occurrence of *dn*DSA and acute rejection. The calibration and discrimination of this score were good in large cohorts of patients treated with the current standard of care.

All previously published scores are computed using only individual factors known before the end of the first year post-transplantation. They are never updated, even if the patient’s prognosis is altered. The performance of these scores is usually evaluated with respect to shorter term graft survival and at a single time point [3–5,10,25]. In this study, we used the non-parametric RSF method which has several advantages compared to regression approaches among which it does not test the goodness of fit of data to a hypothesis, but seeks a model that explains the data [26].

Most of the baseline predictors selected for the calculation of AdGFS, are well-accepted graft failure risk factors [3,4,9,10,27]. Renal function in the first year post-transplantation was found to be predictive of graft survival at 3, 5 [28,29] and 8 years post-transplantation [4]. Proteinuria has also recently been associated with graft failure in a cohort of 1518 patients [30]. The cut-off points for Scr and proteinuria defined in order to maximize time-dependent ROC AUC, are in accordance with previously published values [30]. Comparison between a score calculated with the baseline parameters included in AdGFS (i.e. the variables collected up to one year post-transplantation) and AdGFS (i.e. adding variables collected after one year post-transplantation) demonstrated the added value of taking into account follow-up data beyond one year post-transplantation.

The present study confirmed the deleterious role of donor age and its link with Scr [2,31]. Donor age above 60 years was retained in different donor quality scoring systems [32] and was also associated with graft outcome after acute ABMR [33]. In the present study, two other baseline predictors were identified: Scr cluster and pretransplant NDSA. Longitudinal Scr clusters, assessing the Scr time-profiles along the first year, have never been used before in predictive model of graft failure. Clustering adds information to the use of single or repeated measurement(s) of biological or clinical markers. Herein, it revealed patient subgroups with homogenous Scr time-profiles. This approach is in line with FDA guidance to better differentiate phenotypes of patients (http://www.fda.gov/downloads/Drugs/GuidanceComplianceregulatoryInformation/Guidances/UCM458485.pdf). For future studies, we propose a graphical tool dedicated to allocating new patients in the clusters (S1 Fig).

No previously proposed score takes into account onset of *dn*DSA beyond one year post-transplantation and their impact on graft survival [5,6,34,35]. Our study, finding a cumulative incidence of 9.3% of *dn*DSA and a 24% rate of graft failure at 3 years after the onset of *dn*DSA, is in accordance with previous studies showing a 5-year post-transplantation cumulative incidence of *dn*DSA from 5.5 to 20% [6,23], a 7 to 9% risk of graft failure in the first year after the occurrence of *dn*DSA, and up to 24% of patients with chronic ABMR and renal failure within 3 years post-DSA [6,24].

AdGFS is the first score to include new-onset *dn*DSA to predict graft survival. The inclusion of *dn*DSA requires an adjustable approach since they may appear at any time. AdGFS can be updated during patient follow-up in case of *dn*DSA or acute rejection. *Dn*DSA’s pathogenicity depends on their association with acute rejection, as previously found by Cooper and colleagues [36]. Taking into account *dn*DSA improved survival prediction beyond 5 years post-transplantation in accordance with published works highlighting that graft loss attributable to *dn*DSA occurs several years after their onset [24].

Other factors classically reported to be associated with graft failure [4,27,32,37], such as HLA mismatches, cold ischemia, recipient gender, and immunosuppressive treatments, were not retained in the score because they did not allow a decrease in the error rate in the RSF analysis, and they did not improve the time-dependent ROC AUC. This was explained by their significant association with the retained variables (e.g increased cold ischemia time was associated with Scr clusters).

Contrary to published scores, AdGFS predicted graft failure at different post-transplantation times up to ten years and stratified the patients into four risk groups. Kasiske and colleagues [9] evaluated only the 5 year risk of graft failure and the discriminatory ability of their scores remained modest as highlighted by the authors. In the Kidney Transplant Failure Score, graft failure was evaluated at 8 years post-transplantation and patients were stratified into only two groups [4]. The good results of our external validation in a population different with regards to time of transplantation and standard-of-care supported the robustness of AdGFS.

Assessment of the individual patient’s risk of transplant failure throughout the time after transplantation may be a decisive tool to select the optimal care strategy for the patient. For instance, in the high risk group, specific treatments for *dn*DSA might be questionable regarding the balance between the probability of maintaining a functioning graft and the side effects associated to these treatments.

The main strength of this study lies in the long follow-up and careful monitoring of anti-HLA antibodies using a Luminex® solid-phase assay, even for patients grafted before the year 2000. The database is one of the largest in which the impact of *dn*DSA was analyzed. Our study has some limitations. It included fewer patients than some previously published cohorts and was validated (i) beyond one year of transplantation, (ii) in deceased donors’ grafts, and (iii) in patients without DSA at the time of transplantation. Thereby, AdGFS can be cannot be calculated before one year post-transplantation neither in case of living donor nor in patients with DSA before transplantation.

In conclusion, we propose an adjustable score for risk stratification of graft failure at different post-transplantation times. AdGFS showed good discrimination and could be more useful than scores ignoring onset of *dn*DSA, for decisions regarding more or less intensive surveillance and treatment of the patients.

## Supporting information

S1 Fig Median (10^th^-90^th^ percentiles) serum creatinine profiles over the first year post-transplantation for the three identified clusters, A, B and C. Supplementary individuals can be allocated to a cluster using a graphical approach in which the individual serum creatinine values obtained at each visit are superimposed to the 10^th^-90^th^ percentiles interval of serum creatinine values obtained in the three clusters for the same times. Examples of predicted cluster for three different patients are shown.
(EPS)
